# Sequential Esterification—Diels-Alder Reactions for Improving Pine Rosin Durability within Road Marking Paint

**DOI:** 10.3390/molecules28135236

**Published:** 2023-07-05

**Authors:** Aqsha Aqsha, Haryo Pandu Winoto, Tri Partono Adhi, Sanggono Adisasmito, Yusrin Ramli, Lathifuddin Siddiq, Fauzi Bhakti Pratama, Mohammad Reza Ramdani, Antonius Indarto

**Affiliations:** 1 Department of Chemical Engineering, Institut Teknologi Bandung, Jalan Ganesha 10, Bandung 40132, Indonesia; winotoharyo@gmail.com (H.P.W.); tpadhi@itb.ac.id (T.P.A.); sanggono4444@gmail.com (S.A.); 2 Department of Bioenergy Engineering and Chemurgy, Institut Teknologi Bandung, Jalan Let. Jen. Purn. Dr. (HC), Mashudi No. 1, Sumedang 45363, Indonesia; lathifuddin@students.ac.id (L.S.); fauzibhakti@gmail.com (F.B.P.); mohammadrezaramdani@gmail.com (M.R.R.); 3 Graduate School of Science and Technology, Hirosaki University, 1-Bunkyocho, Hirosaki 036-8560, Japan; ramliyusrin@gmail.com

**Keywords:** binder, Diels–Alder reaction, pine rosin, road marking, sequential esterification, thermoplastic

## Abstract

Pine rosin, which is derived from *Pinus merkusii* resin, a natural product, has demonstrated potential as a road marking binder. Although pine rosin has an excellent shinning property, it has some limitations, such as instability and color change. To tackle these issues, modified rosin has been developed through sequential esterification and Diels–Alder reactions, and it has shown better properties than untreated rosin. In this study, from the evaluation of untreated and treated rosins, the treated rosin showed some improvements, such as a lower acid value and higher stability, as shown by the color consistency during the oxidation test at 150 °C for 24 h in open-air conditions. Additionally, as road marking paint, the modified rosin is blended with blending materials in the range of 18–28 wt.%. The modified rosin has a softening point of 170–210 °C, a hardness of 50–71 HD, and a weight loss of 1.33–5.12 mg during the wearing test. These results are comparable to or better than those of commercially available road marking products.

## 1. Introduction

In the modern era, with the increasing number of vehicle users, ensuring road safety for both pedestrians and drivers has become a concern. To meet the increased demand for road safety regulations, horizontal road paint marking plays an important role. As it always in contact with vehicle wheels, a horizontal road marking must be frequently renewed due to loss of its retro-reflectivity or damage to its layer structure. Heavily trafficked road markings may need to be renewed every 1–2 years. For less heavily used roads, the road markings will need to be renewed every 5–7 years. This process increases the cost of road maintenance and new road marking cases.

One solution to this problem is to provide affordable, durable, and highly visible road marking paint [1,2,3,4]. A critical component in the production of road marking paint is the binder, which is typically made from aliphatic hydrocarbon and rosin-derived products, such as rosin ester and epoxy resins [5,6,7,8]. Aliphatic hydrocarbon-based paint has excellent durability and stability in weathering conditions and is typically used in rural areas [2]. In contrast, rosin-based paint is more resistant to fuel and lubricant oil, making it more suitable for use in urban areas [9]. Furthermore, the binder must meet specific requirements, such as being colorless and durable under any conditions, whether developed from petroleum or bio-based sources [10,11,12].

The use of thermoplastic paint is one potential approach to producing road marking paint, as it is a relatively low-cost product and is easy to use compared to cold-plastic materials. Furthermore, thermoplastic is highly durable, with constant retro-reflectivity compared to other paint types [13,14,15,16]. However, it does require more energy to heat and is sensitive to air temperature [15,16]. Rosin, including pine (*Pinus merkusii*)-based rosin, can be used as a thermoplastic marking paint binder. This environmentally friendly and sustainable material is widely available in Indonesia, China, and Brazil [17,18,19]. In addition, pine rosin is commonly used as an antibacterial agent, ink toner, paint, and coating [19,20,21,22,23,24,25,26]. Given its versatility and renewable source, rosin has great potential as a renewable and sustainable binder in road marking paint. Nonetheless, pine-based rosin suffers from several drawbacks, including a lack of oxidation stability, high acid value, and poor durability [23,24,27,28], due to the carboxylic acid content in pine rosin. Therefore, additional chemical treatments are necessary to equip pine-based rosin with highly durable properties.

In this study, an esterification reaction with glycerol was utilized to convert pine rosin into ester rosin. The reaction between glycerol and rosin will convert carboxylic acids into ester groups that can more stably prevent oxidation reactions with free radicals and oxygen [29,30,31]. Unfortunately, the esterification product still has a high acid value and low resistance to water and high pH conditions [32]. To overcome these limitations, further modification through the Diels–Alder reaction was performed, which transferred hydrogen from hydrolyzed maleic acid at high temperatures to saturate the double bond in the ester rosin. This process eliminated the weaknesses of the raw rosin, producing a highly durable material that could be used as a commercial road marking paint binder. Later on, the mixing evaluation with other components, i.e., filler, plasticizer, and glass bead, was conducted to ensure that the resulting road marking properties are similar to or better than those of commercial products.

## 2. Results and Discussion

### 2.1. Modification of Gum Rosin

#### 2.1.1. Single Esterification Reaction

As previously mentioned, the pine rosin underwent chemical modification via an esterification reaction with ester and maleic anhydride. However, in order to understand the importance of each reaction, the first step of the gum rosin modification was performed via a single esterification reaction. The quality of the modified rosin was evaluated through analysis of the acid value, oxidation stability, and light intensity difference value. The esterification of rosin reduced the acid value from 226 mg-KOH/g (before reaction) to 79–120 mg-KOH/g (after reaction). The high acid value content of the feedstock contributed to the presence of various abietic acids. Unfortunately, these acids were less stable and prone to oxidization in the presence of oxidation agents, i.e., oxygen that was exposed to atmospheric conditions. The oxidation reaction resulted in the product having a darker color. This result is the main reason why either some gum rosin should be protected or the molecules should be converted into more stable ones without changing the main original characteristic of gum rosin, such as shiny appearance, solidity, etc. Esterification, i.e., by adding alcohol or glycerol, is a method that we can use to make the rosin more stable.

**Figure 1:**
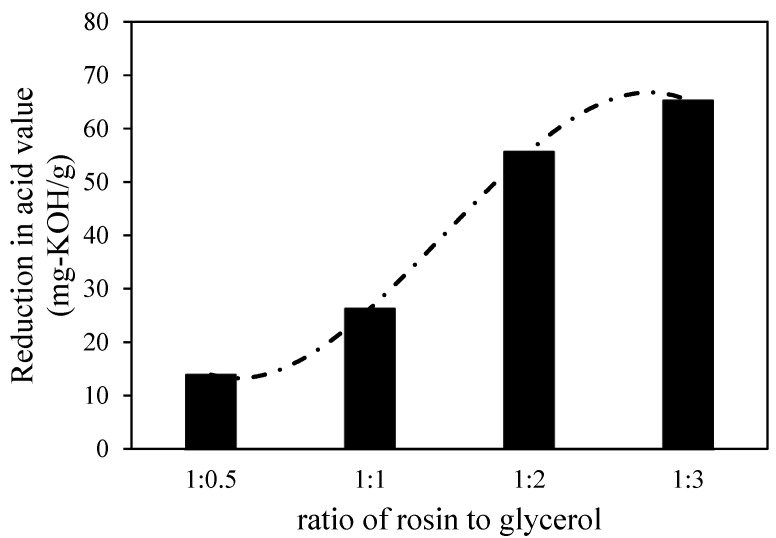
The influence of the ratio of rosin to glycerol on the reduction in the acid number of the resin.

The reduction in acid value is significantly influenced by the amount of glycerol added to the reactor. In general, as shown in Figure 1, when the ratio of rosin to glycerol was increased, the lower acid value was achieved, but the reduction in the acid value from the ratio of rosin to glycerol of 1:2 to 1:3 shows insignificant changes (only 10% increment from 55% to 65%). As the natural property of gum rosin is highly viscous, excess glycerol is required to ensure that the esterification reaction occurred in a shorter time and at a lower temperature. According to overall reaction stoichiometry Equation (1), three moles of pine rosin (representatively shown by abietic acid) reacted with one mole of glycerol in esterification. Due to this fact, the esterification could be completed when the ratio of rosin to glycerol is 1:3. This result means that the formed diacyl and triacyl glycerols as intermediate products can be minimized. As a result, the acid value of treated gum rosin could be reduced significantly. Thus, the chosen operating condition for the next modification involved using a ratio of rosin to glycerol of as much as 1:3.

3 C_20_H_30_O_2_ + C_3_H_8_O_3_ → C_63_H_92_O_6_ + 3 H_2_O (1)

#### 2.1.2. Diels–Alder Reaction

**Figure 2:**
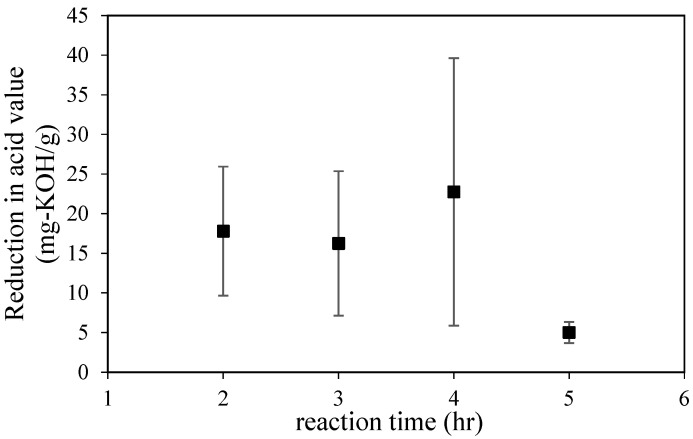
The reduction in acid value via Diels–Alder reaction using maleic anhydride.

Diels–Alder reaction, which is performed using maleic anhydride (rosin fortification reaction), is proposed as another way to reduce the presence of abietic acid and create a more stable product. The advantages of fortified products over unmodified rosin are their wider variety of uses and better quality. For paper-making purposes, fortified rosin can be used in smaller quantities than unfortified rosin. However, compared to glycerol, the maleic anhydride is expensive. However, both components could maintain the original properties of gum rosin. Thus, the reaction was performed at a ratio of rosin to maleic anhydride of 1 to 1 and a temperature of 150 °C. As shown in Figure 2, the highest reduction in acid value was found after a reaction time of 4 h. After 2 and 3 h of the reaction, variations seem to have similar values, and, interestingly, after 5 h of the reaction, the conversion was only 5 mg per KOH/g. Based on the study of Wiyono et al. [33], it seems that the Diels–Alder reaction of gum rosin seems to be a complex reaction that includes many reaction pathways. One of those pathways is the formation of another carboxylic acid compound that will maintain the acid value of the end-product. Additionally, the acid value can also be attributed to the hydrolyzed products that originate from the maleic anhydride, which might occur due to the presence of other acids as side-product of the Diels–Alder reaction. 

According to the resulting data for 2–4 h, the rate of acid value reduction was around 6.6 mg per KOH.g^−1^/h. In contrast, after 4 h of reaction, the reduction rate was 33 mg per KOH/h, which is the optimum value for acid reduction. Furthermore, as mentioned above, the side products of maleic anhydride might contribute to the acid value. As shown in Figure 2, the substantial drop in acid value occurred until 4 h of reaction time; straightforwardly, this result also shows that side-products (other acids from hydrolyzation of maleic acid) might be diminished after 4 h of reaction. The result is similar to the previous result that mentioned that the optimum conversion of abietic acid to levopimaric acid occurred when the reaction time was more than 1 h. However, after a longer reaction time, the ester formation was smaller.

#### 2.1.3. Combined Esterification and Diels-Alder Reaction

In order to obtain the lowest value of acid in the gum rosin, a sequential reaction of esterification and a Diels–Alder reaction were conducted based on the above reaction results. By combining the reaction, the acid value of the final product reached 15–25 mg per KOH/g. This result indicates that content of almost all acid groups could be reduced by >80% through using this technique. 

**Figure 3:**
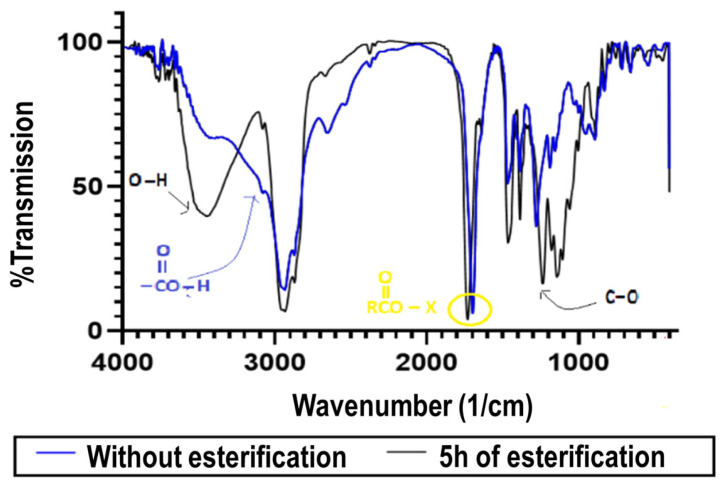
FTIR analysis of modified and unmodified rosins.

In order to ensure that esterification occurred, the raw rosin and spectra of the treated rosin were analyzed using FTIR, as shown in Figure 3. Figure 3 compares the spectra of gum rosin before and after 5 h of reaction using a ratio of rosin to glycerol of 1:3. The main differences are the presence of new wavenumbers at 1700–1725 cm^−1^ and 1735–1750 cm^−1^, which correspond to the carboxylic and esters groups, respectively. Interestingly, the new wavenumber also occurred at 3400–3600 cm^−1^, which is known as O-H stretching. The O-H bond might be a result of the unreacted glycerols placed in a solution of treated pine rosin. Also, the O-H bond might be an effect of the presence of water as a by-product in the esterification process. Moreover, the O-H bond could be evidence of the presence of glyceryl 1-monoabietate as the product stemming from either the esterification of abietic acid with glycerol [24] or the O-H bond of the modified esterification product created via the Diels–Alder reaction.

Moreover, Figure 3 reveals a new range of 1000 to 1400 cm^−1^, which is known as the C-O bond. This new bond offers evidence of the bond between alkyls and oxygen (R-CO-O-R′) in the ester products. This bond could also be considered evidence that esterification was performing effectively. On the other hand, the peak at a wavenumber of 3100 cm^−1^, which was present in the feed sample, was reduced in the product. This result means that the carboxylic acids have been converted into esters following the above reaction mechanism. Hence, based on this result, the sequential esterification and the Diels–Alder reaction could be concluded to have successfully converted the gum rosin into a more stable product.

**Figure 4:**
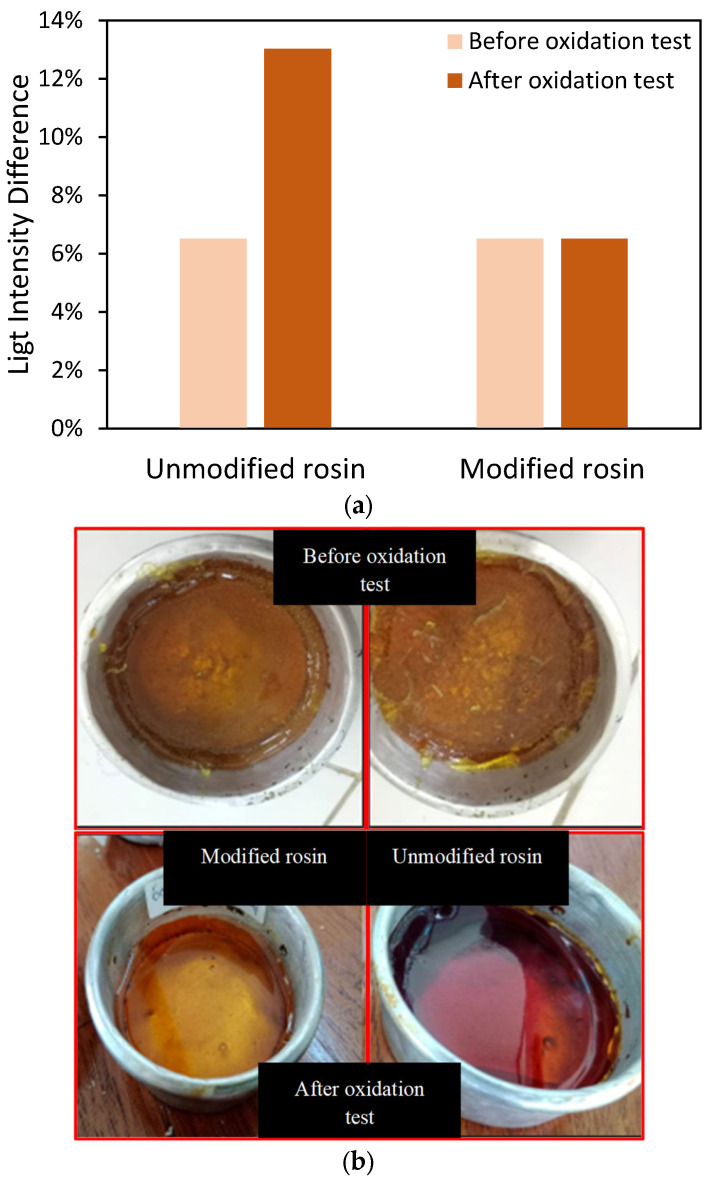
(**a**) Light intensity differences between rosin before and after the oxidation test; (**b**) rosin’s appearance before and after the oxidation test.

Moreover, to check the stability of synthesized gum rosin, the experiment was further checked via a stability test. The sample was heated and exposed to open-air conditions. Figure 4a shows the light intensity of the modified and unmodified rosin samples before and after the oxidation tests. Initially, both samples had similar light intensity values before the oxidation test. However, the light intensity of the unmodified rosin dramatically increased from 6.5 to 13.0% after the oxidation test, indicating a lack of stability. Since the test occurred at high temperatures (150 °C) and in open-air conditions for 24 h, the abietic acid might have been oxidized to other products, and the reaction mechanism was too complex to determine the specific products from abietic acid oxidation [34,35]. On the other hand, the modified resin showed superiority, as its color was not altered by the oxidation process, as in shown Figure 4b. Qualitatively, the color of both rosin samples before the oxidation test was light brown, but after the test, the color of the unmodified rosin became dark brown in color. Hence, the modification also showed that the maleic anhydride successfully saturated the C=C bonds in the rosin [17].

### 2.2. Road Marking Performance Tests

**Figure 5:**
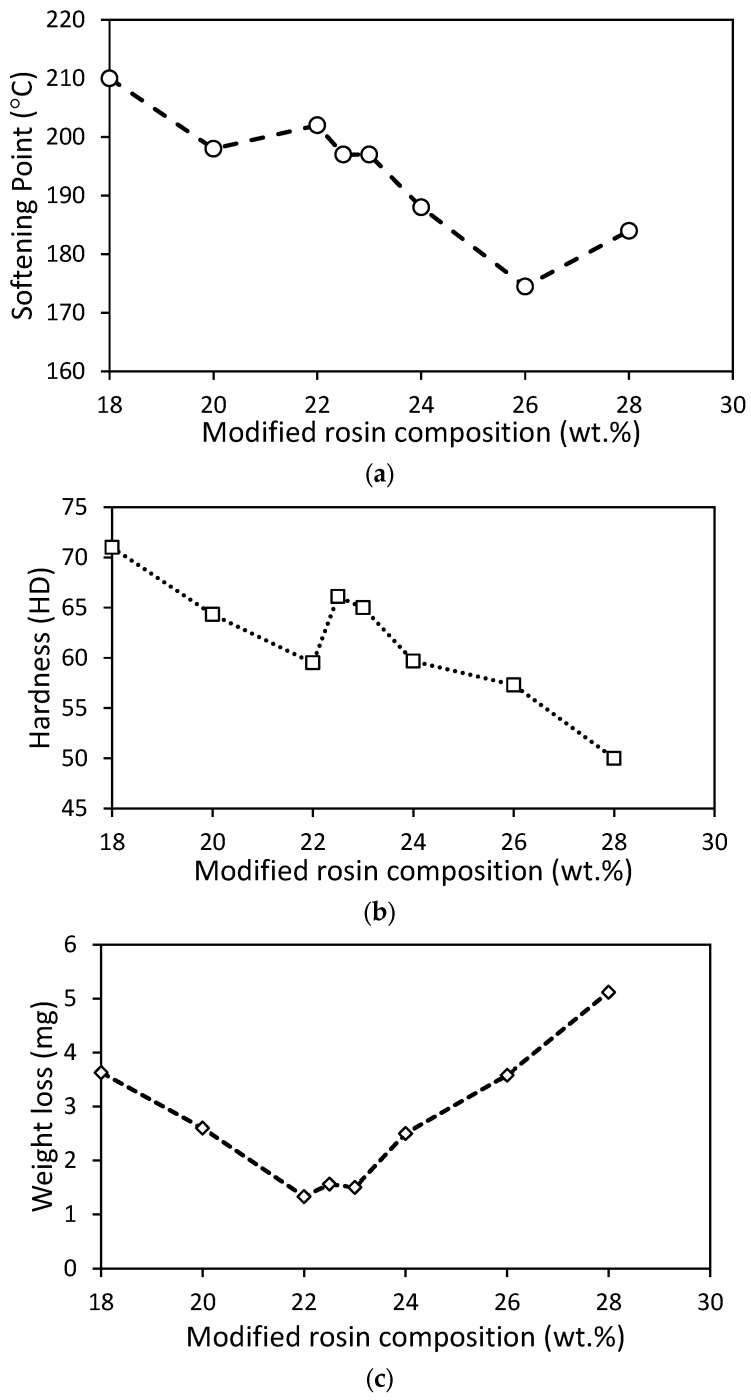
Road marking test results: (**a**) softening point test; (**b**) paint’s hardness test; (**c**) weight loss after wearing test.

In order to evaluate the softening point, each version of the road marking paint product was tested using the ring and ball method. The results indicated a range of softening points between 174 and 210 °C. Figure 5a shows that an increase in the rosin composition resulted in a decline in the softening point. It is worth noting that the desired softening point should be above 100 °C, with a preference for it to be above 130 °C. This preference exists because a higher softening point can increase the cohesive strength [36,37] of the binder, making the road marking paint suitable for high-temperature regions [38]. Additionally, conventional Indonesian road marking paint was tested, and the softening point was found to be 170 °C, which is the value nearest to that of the road marking paint at 26% rosin composition.

Generally, a lower rosin composition resulted in a higher hardness index, as the characteristics of CaCO_3_ became dominant. Here, CaCO_3_ was used as the hardness control, since the CaCO_3_ (limestone) is known to be a soft stone similar to marble. Consequently, the hardness of the paint decreased with an increase in rosin content. The hardness of the paint is shown in Figure 5b, ranging from 50 to 71 HD. According to Smooth-On Inc [39], the hardness values were recorded in hard-to-extra-hard materials, such as shopping cartwheels and hard hats. On the other hand, the hardness of commercial thermoplastic marking paint designed by Landscapus Inc (Beijing, China) [40] was disclosed as being in the range of 45 to 75 HA (Shore A), which is in the medium-soft and medium-hard ranges, such as pencil eraser and tire tread [39]. Nevertheless, Table 1 presents the hardness of Indonesian road marking paint as being as high as 71 HD (extra hard). In this study, the paint made of 18% rosin composition was recorded as having the same value as Indonesian conventional paint, followed by the 22.5% rosin paint.

In addition, Figure 5c illustrates weight loss as an assessment for homogeneity and compatibility between the rosin and blending materials. The lowest weight loss occurred when the rosin composition was between 22 and 23 wt.%. In comparison, when the unmodified rosin paint was evaluated using the same method, the weight loss from the wear resistance test was 8.6 mg/kg, while that of the modified resin was only 1.33–5.12 mg/kg. Compared to conventional paint, the weight loss of paint from 22–23% rosin was near to or almost the same (1.33 to 1.56 mg/kg) as that of the conventional paint (1.51 mg/kg).

**Figure 6:**
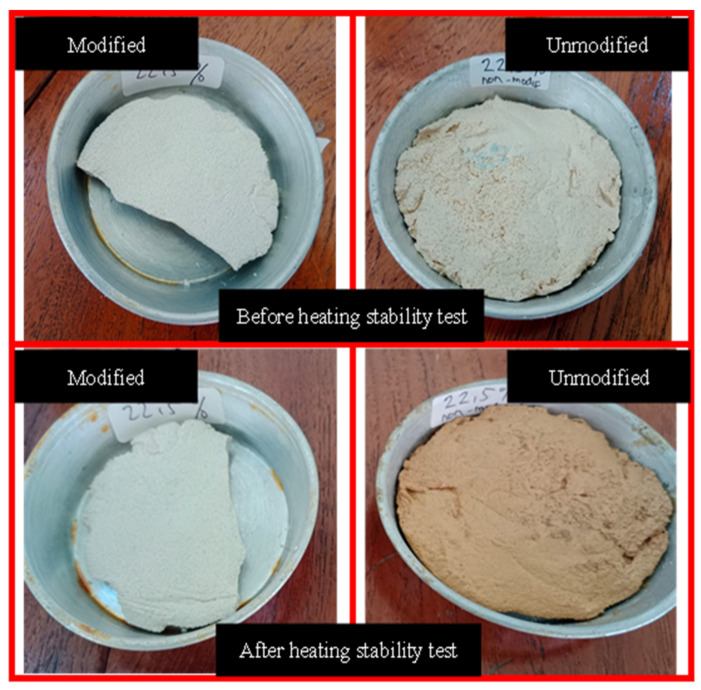
Heating stability test performed on modified and unmodified rosin road markings.

In brief, considering the performance test, the 22.5% modified rosin version was chosen as the best result, since the characteristics were almost the same as those of conventional paint. The characteristics of 22.5% modified rosin paint, 22.5% unmodified rosin paint, and conventional paint are described in detail in Table 1. The table presents a comparison between the quality of road marking paint using modified rosin and unmodified rosin at the same content (22.5 wt.%) as that of the paint’s binder. The results demonstrate that the softening point of the modified rosin paint is higher than that of the unmodified paint, which is attributed to the Diels–Alder reaction between rosin ester and maleic anhydride [17]. Maleic anhydride is known to be a softening point improver material. Additionally, the hardness of the modified rosin paint is higher than that of the unmodified paint, and the modified rosin has a hardness closer to that of the conventional product. Moreover, the weight loss of the modified rosin paint in the wearing test is comparable to that of the conventional product. Furthermore, Figure 6 presents a comparison between the heat stability tests of the modified and the unmodified rosin paints. The figure reveals that the modified rosin paint is more stable, since its color is not significantly influenced by heating. 

**Figure 7:**
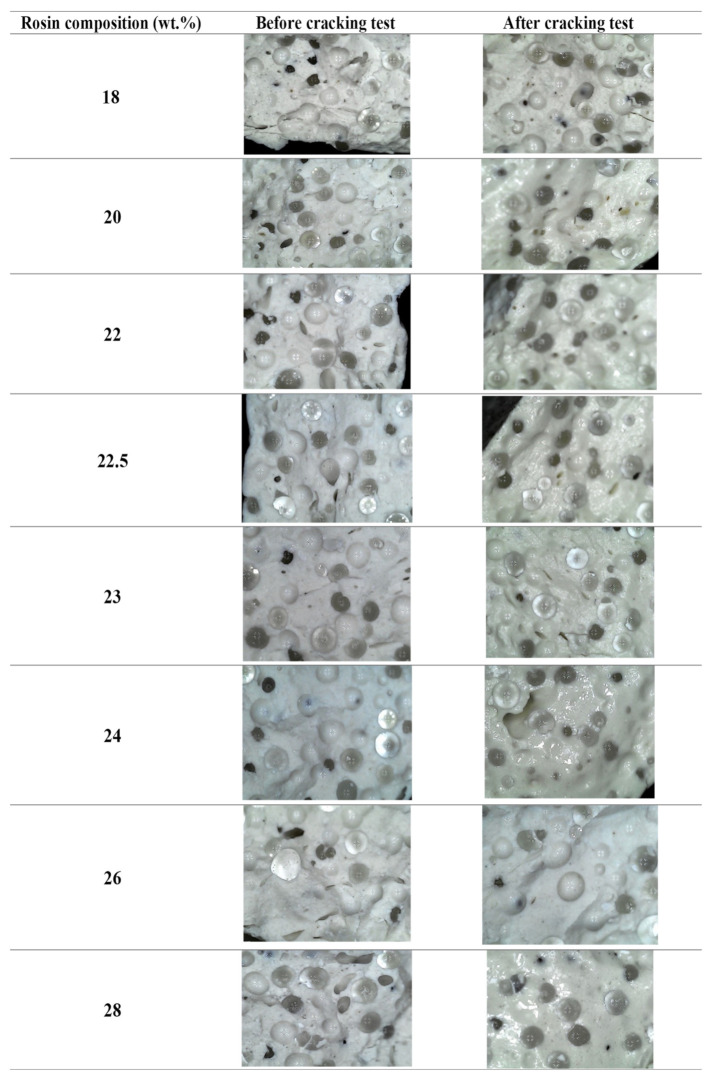
Microscopic image of cracking test at 40 mm magnification.

Based on the results presented in Figure 7, it can be concluded that all versions of the road marking paint were able to withstand heating at 150 °C for 24 h without exhibiting any cracks. This result indicates that the interaction between the binder and blending materials was compact [5] and able to withstand high temperatures, making it suitable for use in hot climates. Additionally, Figure 7 confirms there were no defects observed in the road markings, as well as that the glass beads were effective in providing reflectivity. Overall, the modified pine rosin binder demonstrated versatility across different rosin compositions, further highlighting its potential as a reliable binder for creating road marking paint.

## 3. Materials and Methods

### 3.1. Materials

In this study, pine rosin was used as the binder of the thermoplastic road marking. Pine rosin was obtained from the Perhutani Pine Chemical Industry (PPCI) in Pemalang, Indonesia. As mentioned before, the pine rosin was treated before it was utilized as a binder to improve its properties. Other components, such as talc, glass beads, CaCO_3_, TiO_2_, wax, and plasticizer, were purchased in technical grade. Moreover, glycerol and maleic anhydride were used as blending–modification materials. Other materials were nitrogen, potassium hydrogen phthalate (KHP), potassium hydroxide (KOH), toluene, phenolphthalein (PP), and deionized water. All reagents were used in technical grade and as received without further purification.

### 3.2. Rosin Modification

To start the modification, the rosin was melted at 150 °C on a hotplate in a three-neck flask under nitrogen flow to prevent oxidation from reacting with oxygen. Firstly, glycerol was added to the rosin in ratios of 1:0.5, 1:1, 1:2, and 1:3 (w-rosin/w-glycerol), and the esterification reaction was carried out at 250 °C for 5 h to enhance stability [29,41]. Additionally, the effect of reaction time was studied by varying the reaction time by as much as 2, 3, 4, and 5 h and using a rosin/glycerol ratio as much as 1:3. By evaluating the acid value, the least acid value variation underwent further analysis, Fourier-transform infrared spectroscopy (FTIR) was used to scrutinize carboxylic acids and esters groups in untreated and treated rosin. Also, the least acid value variation was chosen as the main operating condition.

After the esterification process was completed, maleic anhydride was introduced into the flask at a ratio of 1:0.5, 1:1, and 1:2 with rosin, and the Diels–Alder reaction was performed at 150 °C for 1–3 h. Later on, the chosen operating conditions for maleic anhydride fortification were set at a ratio of 1:1, and reaction time was set at 1 h. The addition of maleic anhydride enhances oxidation stability by saturating double bonds in the rosin through the Diels–Alder reaction [17].

### 3.3. Blending Methods

The modified rosin was used as a binder and blended with various materials, including talc, glass beads, CaCO_3_, TiO_2_, wax, and plasticizer. The percentage of modified rosin and CaCO_3_ used in the blend ranged from 18–28% and 36–26%. On the other hand, talc, glass beads, TiO_2_, wax, and plasticizer were added at fixed percentages of 2%, 30%, 10%, 2%, and 2%, respectively.

**Figure 8:**
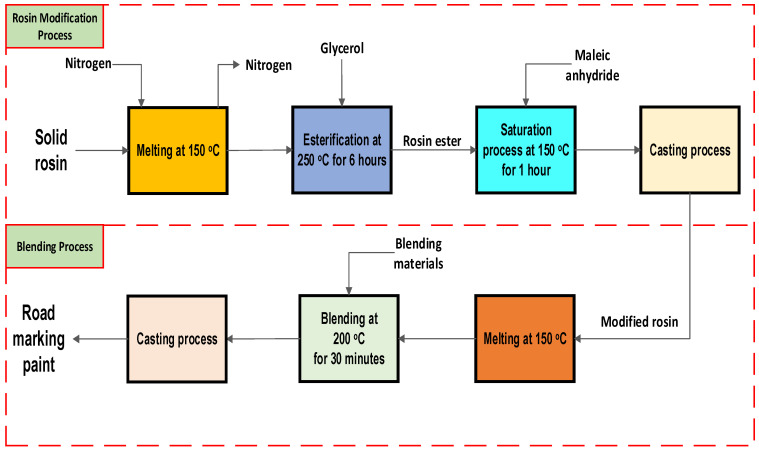
Modification and blending processes.

To begin the blending process, the modified rosin was melted at 150 °C with constant stirring at 150 rpm. The temperature was then raised to 200 °C, and the blending materials were added and mixed for 30 min until a homogeneous mixture was achieved. The final product was then poured into an aluminum plate and tested against a product made with 22.5% unmodified rosin. Figure 8 summarizes the modification and blending processes.

### 3.4. Analysis Methods

In this study, we measured the acid value, oxidation stability, and light intensity difference of the resin before and after the reaction. The acid value was determined through an acid-base titration that used KOH as the titrant and phenolphthalein as the indicator. Equation (2) was used to calculate the acid value based on the amount of KOH used in the titration.

(2) $$\mathrm{acid}\;\mathrm{value}\;\left(\mathrm{mg}-\mathrm{KOH}/g\right)=\frac{56.1\times \;V\;\times \;N}{m}$$

where V = volume of used KOH (mL), N = normality of KOH in ethanol (N), and m = sample weight (g).

For the oxidation stability test, the rosin was heated in an oven at 150 °C for 24 h in open-air conditions, and the stability was measured visually by comparing the color of the rosin before and after the heating treatment. As the color of the rosin should be maintained in a clear solution, the intensity of light passing through the liquid rosin was measured using a lux meter (JRLGD, LX1010B with a measuring range of 0 to 50,000 lux), which was then used to calculate the light intensity value via comparison with water as a reference, as shown in Equation (3).

(3) $$\mathrm{light}\;\mathrm{intensity}\;\mathrm{difference}\;\left(\%\right)=\frac{A-B}{A}\times 100\%$$

where A = reference light intensity (lux), and B = sample light intensity (lux).

Higher light intensity difference values mean that the color of the sample was becoming darker.

In order to evaluate the performance of the road marking, several tests were conducted, such as softening point analysis, wear resistance test, heat stability analysis, hardness analysis, and cracking resistance test. The softening point analysis was performed using a common ring and ball method. The wear resistance test involved using an electric abrasion method at a speed of 5047 rpm for 1 min. Later on, the difference in the weight of the sample before and after the abrasion test will reflect the durability of the sample during the abrasion process.

Heat stability analysis was conducted by heating the paint to 150 °C for 24 h exposed to the atmospheric air to evaluate the color stability, as well as the layer structure of the sample. Durometer Shore D was used to measure the hardness of the road marking samples for 30 s. Durometer Shore D has the capability to measure semi-rigid plastics and hard plastics, and the product used in this study was thermoplastic material. Lastly, the cracking resistance test was performed using a digital microscope with 1600× of magnification to observe any cracks in the samples before and after the stability test.

## 4. Conclusions

In summary, this study demonstrated the effectiveness of modifying pine rosin through sequential esterification and Diels–Alder reactions. This method successfully converted the raw gum rosin into more stable products, as evidenced by the new O-H bond and C-O bond, which indicated that the reactions had been modified in the raw rosin. The modified rosin was synthesized with a reaction with glycerol and maleic anhydride, which achieved >85% of reaction conversion (treated rosin acid value of 15–25 mg-KOH/g). Additionally, the modified rosin showed improvements in characteristics such as oxidation stability, which can enhance the quality of road marking materials. On the other hand, the road marking paint made from 22.5 wt.% modified rosin showed better wear resistance than conventional products, although its hardness is slightly lower. However, the modified rosin paint had a higher softening point, making it suitable for use in hot regions. The cracking test results also demonstrated that the road marking paint made from modified rosin had good sturdiness. Overall, this study suggests that modified rosin has the potential to be used as a road marking binder, promoting sustainability and protecting our infrastructure.

## Figures and Tables

**Table 1:** Comparison between modified and unmodified rosin in road marking paint with a conventional paint test result.

Parameter	Road Marking Parameter		
	Modified Rosin	Unmodified Rosin	Commercial
Softening point (°C)	197	158	170
Hardness (HD)	66	60	71
Weight loss (mg)	1.56	8.60	1.51

## Data Availability

No new data were created or analyzed in this study. Data sharing is not applicable to this article.
